# Synthesis of New Five Coordinated Copper(II) and Nickel(II) Complexes of L-Valine and Kinetic Study of Copper(II) with Calf Thymus DNA

**DOI:** 10.1155/MBD.2002.81

**Published:** 2002

**Authors:** Aijaz Ahmad Tak, Farukh Arjmand, Sartaj Tabassum

**Affiliations:** 1 Department of Chemistry Aligarh Muslim University Aligarh 202002 India

## Abstract

Five coordinated novel complexes of ${\text{Cu}}^{\text{II}}$ and ${\text{Ni}}^{\text{II}}$ have been synthesized from benzil and 1,3- diaminopropane-${\text{Cu}}^{\text{II}}$/${\text{Ni}}^{\text{II}}$ complex and characterized by elemental analysis, i.r., n.m.r., e.p.r, molar conductance and u.v-vis, spectroscopy. The complexes are ionic in nature and exhibit pentaeoordinated geometry around the metal ion. The reaction kinetics of ${\text{C}}_{25}{\text{H}}_{36}{\text{N}}_{\text{5}}{\text{O}}_{2}\text{CuCl}$ with calf thymus DNA was studied by u.v-vis, spectroscopy in aqueous medium. The complex after interaction with calf thymus DNA shows shift in the absorption spectrum and hypochromicity indicating an intercalative binding mode. The ${\text{K}}_{\text{obs}}$ values have been calculated under pseudo-first order conditions. The redox behaviour of complex ${\text{C}}_{25}{\text{H}}_{36}{\text{N}}_{\text{5}}{\text{O}}_{2}\text{CuCl}$ in the presence and in the absence of calf thymus DNA in the aqueous solution has been investigated by cyclic voltammetry. The cyclic voitammogram exhibits one quasi-reversible redox wave corresponding to ${\text{Cu}}^{\text{II}}$/${\text{Cu}}^{\text{I}}$ redox couple with ${\text{E}}_{1/2}$ values of -0.377 and -0.237 V respectively at a scan rate of 0.1V${\text{s}}^{\text{-}1}$ .On interaction with calf thymus DNA, the complex ${\text{C}}_{25}{\text{H}}_{36}{\text{N}}_{\text{5}}{\text{O}}_{2}\text{CuCl}$ exhibits shifts in both ${\text{E}}_{\text{p}}$ as well as in ${\text{E}}_{1/2}$ values, indicating strong binding of the complex to the calf thymus DNA.


### Synthesis of New Five Coordinated Copper(II) and Nickel(II) Complexes of L-Valine and Kinetic Study of Copper(II) with Calf Thymus DNA

Aijaz Ahmad Tak, Farukh Arjmand and Sartaj Tabassum*

*Department of Chemistry, Aligarh Muslim University, Aligarh- 202002 India*
*E-mail: tsartaj62 @ yahoo.com.*

### ABSTRACT

Five coordinated novel complexes of $Cu^{II}$ and $Ni^{II}$ have been synthesized from benzil and 1,3-diaminopropane-$Cu^{II}$/$Ni^{II}$ complex and characterized by elemental analysis, i.r., n.m.r., e.p.r, molar conductance and u.v-vis. spectroscopy. The complexes are ionic in nature and exhibit pentacoordinated geometry around the metal ion. The reaction kinetics of $C_{25}H_{36}N_5O_2CuCl$ with calf thymus DNA was studied by u.v-vis. spectroscopy in aqueous medium. The complex after interaction with calf thymus DNA shows shift in the absorption spectrum and hypochromicity indicating an intercalative binding mode. The $k_{obs}$ values have been calculated under pseudo-first order conditions. The redox behaviour of complex $C_{25}H_{36}N_5O_2CuCl$ in the presence and in the absence of calf thymus DNA in the aqueous solution has been investigated by cyclic voltammetry. The cyclic voltammogram exhibits one quasi-reversible redox wave corresponding to $Cu^{II}/Cu^{I}$ redox couple with $E_{1/2}$ values of -0.377 and -0.237 V respectively at a scan rate of $0.1 Vs^{-1}$. On interaction with calf thymus DNA, the complex $C_{25}H_{36}N_5O_2CuCl$ exhibits shifts in both $E_p$ as well as in $E_{1/2}$ values, indicating strong binding of the complex to the calf thymus DNA.

### INTRODUCTION

Copper is widely distributed in the biological systems. Complexes of $Cu^{II}$ are capable of interaction with nucleic acids and have been intensively studied /1-3/. Sigman *et al.* have proved $Cu(Phen)_2^+$ to be an efficient nuclease which can be employed for understanding the DNA binding mechanism in DNA complexes /4-6/. Artificial nucleases have been proved to be an efficient tool for the footprinting and sequence specific targeting of nucleic acids /7,8/. Despite the intense study, the exact mode of binding remains unknown /9-11/. Current researchers are developing new applications and finding new ways to design more efficient and

* Author for correspondence useful catalysts for DNA cleavage. $Cu^{II}$-L-histidine is one of the well defined complexes which has been demonstrated to cleave plasmid DNA at physiological pH and temperature /12/. Metal ion-L-histidine interaction (with $Cu^{II}$, $Ni^{II}$, $Zn^{II}$ etc) has also been well demonstrated and can be probed for other applications as well, viz, protein purification /13-15/. In continuation to our earlier report on $Co^{II}$ complexes of five coordinated amino acid porphyrin interaction with calf thymus DNA, we have designed new $Cu^{II}$/$Ni^{II}$-L-valine complexes to understand the mechanistic pathway of DNA binding at physiological pH.

Amino acids are the basic structural units of protein and reports on different amino acid residues reveal that they are capable of controlling the binding and reaction of the $Cu^{II}$ complexes on DNA /16,17/. These amino acids play an important role at and around the active sites of various metalloproteins including regulating the redox potential of the metal ion, arranging the specific geometry of the metal coordination site and restricting the conformation of the peptide chain /18/. Thus amino acids interaction promotes specificity of the molecule to the active site including regulating the redox properties, which we have demonstrated through cyclic voltammetric data. In this paper we report the synthesis of new valine complexes of type $C_{25}H_{36}N_5O_2CuCl$/$C_{25}H_{36}N_5O_2NiCl$ derived from benzil and 1,3-diaminopropane$Cu^{II}$/$Ni^{II}$ complex and the interaction of $Cu^{II}$ complex with calf thymus DNA.

### EXPERIMENTAL

All experiments involving interaction of complex with DNA were carried out in aqueous solution with varying concentrations of CTDNA ($10 \times 10^{-5}$, $12 \times 10^{-5}$, $14 \times 10^{-5}$, $16 \times 10^{-5}$ $18 \times 10^{-5}$ mol $dm^{-3}$). The CTDNA concentration was determined by absorption spectrophotometry. Doubly distilled water was used throughout. The stock solution of calf thymus DNA was prepared by dissolving it in 10 ml tris HCl buffer at pH 7 and dialyzing against the same buffer for 48 h. The solution gave a ratio of >>1.8 at A 260/280, indicating that calf thymus DNA was free from protein /19/. The concentration of calf thymus DNA was determined by monitoring the u.v. absorbance at 260 nm using $\Sigma 260 = 6600\ cm^{-1}$.The stock solution was stored at –20°C. $NiCl_2$, $CuCl_2$, (hydrated) (BDH), 1,3-diaminopropane (Merck), L-Valine (Sisco) and Benzil (Fluka) were used as received. and calf thymus DNA was obtained from Sigma. Microanalysis of samples was obtained on Carlo Erba analyzer Model 1106. I.r. spectra (200-4000) $cm^{-1}$ were recorded on Carl Zeiss Specord M 80 spectrophotometer in nujol mulls. The electronic spectra were recorded on a Systronic119 spectrophotometer (ESP-300) and n.m.r. spectra on an amx 500 instrument. Cyclic voltammetry measurements were recorded on a CH instrument electrochemical analyzer. High purity $H_2O$/MeOH (95:5) was employed for the cyclic voltammetric (c.v) studies with 0.4M $KNO_3$ as supporting electrolyte. A three electrode configuration was used, comprised a Pt microcylinder as working electrode, Pt wire as auxillary electrode and Ag/AgCl as reference electrode. Experiments were carried out at room temperature.

### Synthesis of Complex $C_6H_{20}N_4CuCl_2$

To the solution of the copper chloride (1.71 g, 10mmol) in MeOH (50 $cm^{-3}$) was added 1,3-diaminopropane (1.70 g, 10mmol) in 1:2 molar ratio. A blue precipitate was obtained, washed with ether and dried in vacuo. Similarly $Ni^{II}$ complex was also synthesized

### Synthesis of Complex $C_{20}H_{26}N_4CuCl_2$

To a solution of benzil (0.210 g, 1mmol) in MeOH (50 $cm^{-3}$) was added $C_6H_{20}N_4CuCl_2$ complex (0.282 g, 1mmol) in 1:1 molar ratio. The resulting solution was boiled to reflux for *ca.* 20 h, while dark green precipitate was obtained, which was washed thoroughly with ether and dried in vacuo.

### Synthesis of Complex $C_{25}H_{36}N_5O_2CuCl$

To a hot solution of L-valine (0.117 g, 1mmol) in MeOH (50 $cm^{-3}$) was added the complex $C_{20}H_{26}N_4CuCl_2$ (0.456g, 1mmol) in DMF. The solution was boiled to reflux for *ca.* 36 h and further concentrated. The solution was then allowed to cool and left overnight in the refrigerator. Blue coloured complex was obtained, filtered, washed with ether and dried in vacuo. $Ni^{II}$ complex was also prepared in a similar way.

**Table 1**

Analytical data

| Complex | Colour | M.P. °C | Yield % | % Analytical Found/Calcd. | | |
|---|---|---|---|---|---|---|
| | | | | C | H | N |
| 1) $C_{20}H_{26}N_4CuCl_2$ | Dark green | 165 | 75 | 52.72 (52.63) | 5.79 (5.70) | 12.39 (12.28) |
| 2) $C_{20}H_{26}N_4NiCl_2$ | Dark red | 180 | 72 | 53.20 (53.09) | 5.81 (5.75) | 12.45 (12.38) |
| 3) $C_{25}H_{36}N_5O_2CuCl$ | Blue | 260(d) | 60 | 56.08 (55.97) | 6.80 (6.71) | 12.23 (13.05 |
| 4) $C_{25}H_{36}N_5O_2NiCl$ | Green | 265(d) | 58 | 56.47 (56.39) | 6.82 (6.76) | 13.26 (13.15) |

### RESULTS AND DISCUSSION

### I.R. Spectra

The most prominent bands in the spectra of the complexes are listed in Table 2. The infrared spectrum of the complex $C_{20}H_{26}N_4CuCl_2$ is devoid of absorption characteristic of the -C=O group. A strong band at *ca.*1590 $cm^{-1}$ assigned to the ν(C=N) vibration indicates the formation of the $C_{20}H_{26}N_4CuCl_2$ complex /20/. A sharp band at 3290 $cm^{-1}$ was assigned to the ν(N-H) stretching absorption /21/. The alkyl $CH_2$ group show characteristic stretching absorption bands in the region 2950, deformation bands at 1478 and rocking modes at 715 $cm^{-1}$ respectively. The phenyl groups show ν(C-H) stretching at 3025 $cm^{-1}$ and ν(C=C) stretching at 1520 $cm^{-1}$ respectively. The presence of the bands at 425-445 $cm^{-1}$ region in all the complexes corresponds to the ν(M-N) vibrations /22/. The free amino acid shows two bands at 1610-1660 and 1395-1430 $cm^{-1}$ region

**Table 2**

I.R. Spectral Data ($cm^{-1}$)

| Complex | νC=N | νC=O | νNH | $\nu CH_2$ | νM-N | νM-O |
|---|---|---|---|---|---|---|
| 1) $C_{20}H_{26}N_4CuCl_2$ | 1590 | -- | 3290 | 2950 | 425 | -- |
| 2). $C_{20}H_{26}N_4NiCl_2$ | 1594 | -- | 3293 | 2945 | 420 | -- |
| 3). $C_{25}H_{36}N_5O_2CuCl$ | 1598 | 1640 | 3289 | 2947 | 430 | 375 |
| 4). $C_{25}H_{36}N_5O_2NiCl$ | 1592 | 1630 | 3296 | 2951 | 445 | 395 |

corresponding to the antisymmetric and symmetric ($COO^-$) stretching vibrations, respectively. On complexation, these bands shift to lower and higher wavenumber respectively, indicating that the amino carboxylate group is involved in the complex formation. The symmetric ν($COO^-$) stretching band appears at 1350 $cm^{-1}$ due to the coordination of carboxylic group of amino acid to the metal ion through oxygen. This is further supported by the appearance of ν(M-O) band in the 375-395 $cm^{-1}$ region respectively which confirms the coordination of the amino acid through oxygen /23/. The complex $C_{25}H_{36}N_5O_2CuCl/C_{25}H_{36}N_5O_2NiCl$ exhibits characteristic stretching absorption bands in the region 2947 $cm^{-1}$, deformation band at 1480 and rocking mode at 713-720 $cm^{-1}$. On the basis of the i.r. spectral data, we propose the coordination environment of the complexes as given in scheme 1.

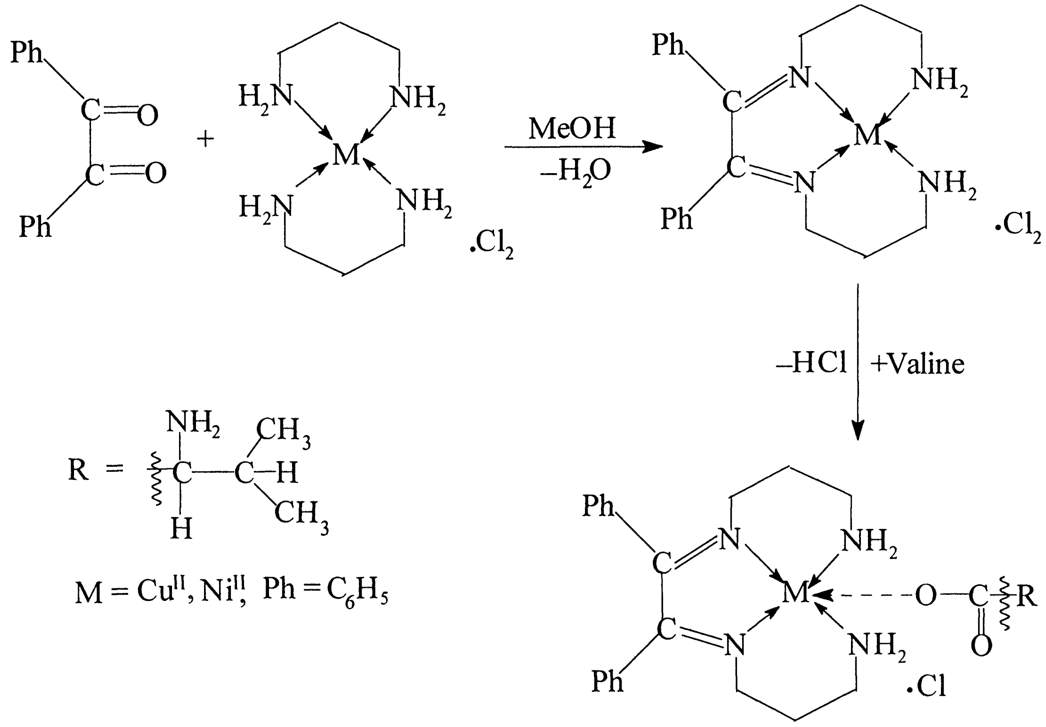

Scheme 1.

### E.P.R. Spectra

The e.p.r. spectrum of the complex $C_{25}H_{36}N_5O_2CuCl$, recorded at room temperature, shows signals for $g_{II}$ and $g_{\perp}$ at 2.25 and 2.07 respectively, which is consistent with the square pyramidal geometry around $Cu^{II}$ ion. The $Cu^{II}$ exhibits $d_{x^2-y^2}$ ground state with $g_{II} > g_{\perp} > 2.0$ which is the most common ground state of these compounds /24/.

### Electronic Spectra

The electronic spectrum of $C_{25}H_{36}N_5O_2NiCl$ recorded in MeOH shows bands at 610 nm and 360 nm which could be assigned to the $^3B_1 \rightarrow {}^3E$ (F) and $^3B_1 \rightarrow {}^3A_2$, $^3E$(P) transitions respectively /25/. These values are consistent with pentacoordinated environment around $Ni^{II}$ /26,27/. The electronic spectrum of $C_{25}H_{36}N_5O_2CuCl$ complex recorded in MeOH shows a broad band at 645nm, which is due to the d – d transition assigned to the pentacoordinate geometry around $Cu^{II}$ ion /28/.

### N.M.R Spectra

In order to arrive at the correct structure of the complexes $C_{20}H_{26}N_4NiCl_2$ and $C_{25}H_{36}N_5O_2NiCl$, $^1H$ and $^{13}C$ n.m.r. spectra have been carried out. The $^1H$ n.m.r. spectrum of the $C_{20}H_{26}N_4NiCl_2$ recorded in DMF shows a multiplet at 7.51–7.93 ppm, due to the phenyl protons. The multiplet due to $CH_2$ protons and $CH_3$ protons was observed at 3.70-3.80 ppm. and 0.62-1.80 ppm respectively. The primary amine proton signal was observed at 5.0 ppm. In the complex $C_{25}H_{36}N_5O_2NiCl$, the absence of carboxylic proton signal of the amino acid confirms coordination to the metal through oxygen atom of carboxylic group. There was no major shift in the δ values after coordination of amino acid to the metal. The primary amine signal appears at 4.90 ppm /29/ and $CH_2$ proton signal at 3.62-3.74 ppm respectively. The mutliplet due to the phenyl protons appears at 7.17-7.43 ppm.

**Table 3**

$^1H$ n.m.r. data δ (p.p.m.)

| Complex | NH(m) | $CH_2$(m) | Ar (m) | $CH_3$(m) |
|---|---|---|---|---|
| 1) $C_{20}H_{26}N_4NiCl_2$ | 4.8-5.0 | 3.7-3.8 | 7.5-7.9 | 0.6-1.8 |
| 2) $C_{25}H_{36}N_5O_2NiCl$ | 4.9-5.0 | 3.6- 3.7 | 7.1-7.4 | 0.5-1.6 |

**Table 4**

$^{13}C$ n.m.r. data (p.p.m.)

| Complex | Phenyl carbon | C=N | C=O | $-CH_2-$ | $-CH_3-$ | Ar-C= |
|---|---|---|---|---|---|---|
| 1) $C_{20}H_{26}N_4NiCl_2$ | 108-124 | 149.6 | 160.1 | 38.2 | 20.1 | 108.6 |
| 2) $C_{25}H_{36}N_5O_2NiCl$ | 120-127 | 142.0 | 165.4 | 39.1 | 22.3 | 109.7 |

### Redox Behaviour

The cyclic voltammetric studies provide an insight to the calf thymus DNA binding. The cyclic voltammogram recorded for the complex $C_{25}H_{36}N_5O_2CuCl$ in $H_2O$/ MeOH (95:5) at a scan rate of $0.1 Vs^{-1}$ reveals one electron quasireversible CuII/I wave with $E_{1/2}$ values as –0.377V and 0.237V respectively (Fig.1). The ratio of anodic to cathodic peak currents is ~ 1. At different scan rates the voltammogram do not show any major change (Fig. 2). For a reversible wave $E_p$ is independent of scan rate and $I_p$ (as well as the current at any point of the wave) is proportional to the $v^{1/2}$ /30/. The $\Delta E_p$ value is larger than the Nernstian value observed for the one-electron transfer couple. Large peak width for one electron couple $Cu^{II} \longrightarrow Cu^{I}$ in these complexes is not an uncommon observation /31/. This is due to the reorganization of the coordination sphere during the electron transfer and has been observed in a number of the copper complexes as well /32/. On addition of calf thymus DNA, the complex experiences a shift in $E_{1/2}$ as well as in $E_p$ values. The ratio of $I_{pa}/I_{pc}$ for the bound complex decreases (0.66), suggesting that calf thymus DNA is bound strongly to the complex. In addition to the changes in the formal potential, the voltammetric peak decreases upon addition of calf thymus DNA to the complex. The decrease in the current is due to the diffusion of the equilibrium mixture of free and DNA-bound metal complex to the electrode surface /33/.

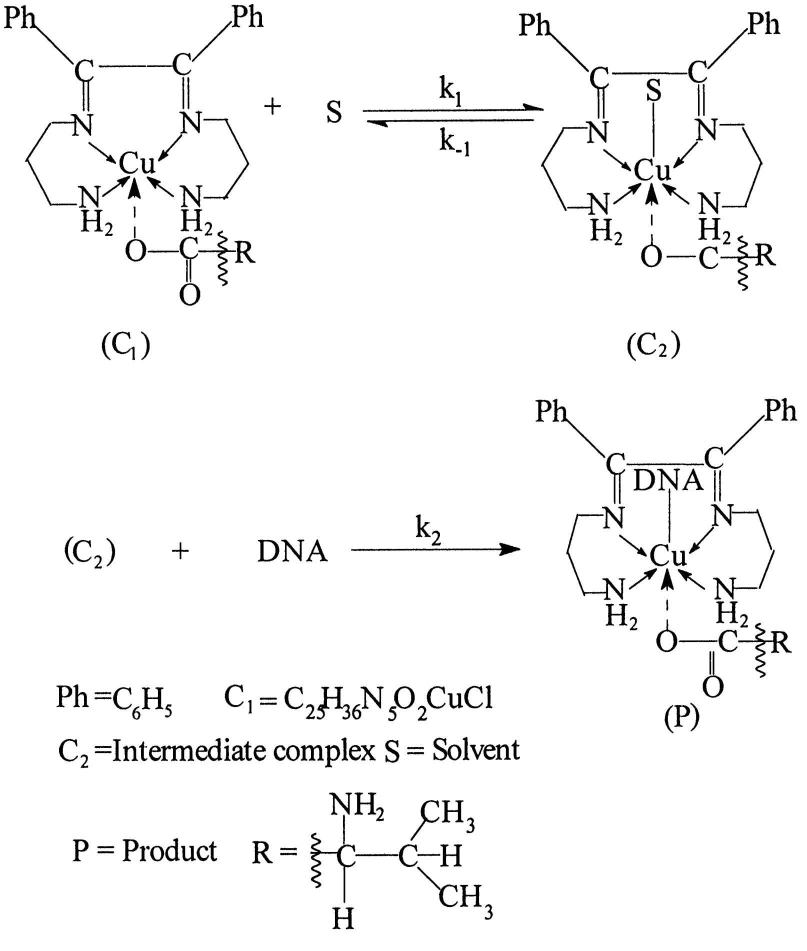

Scheme 2.

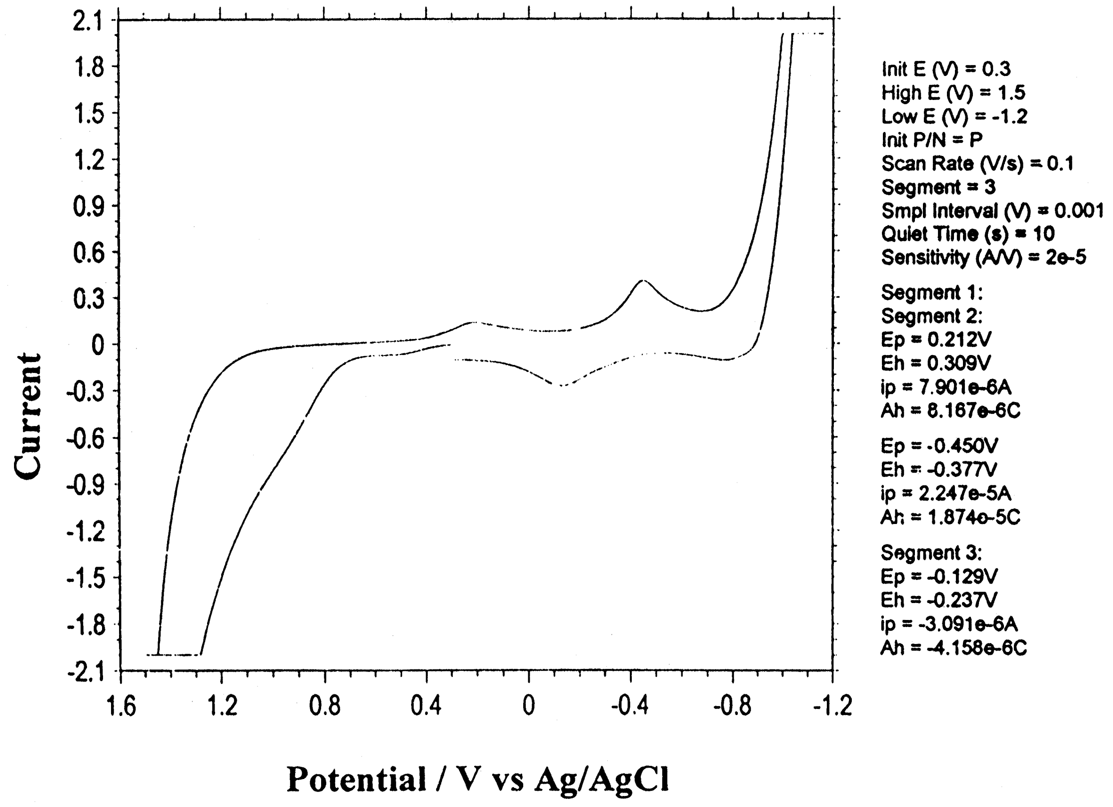

**Fig. 1:** Cyclic voltammogram of complex $C_{23}H_{36}N_5O_2CuCl$ at scan rate of 0.1 v/s.

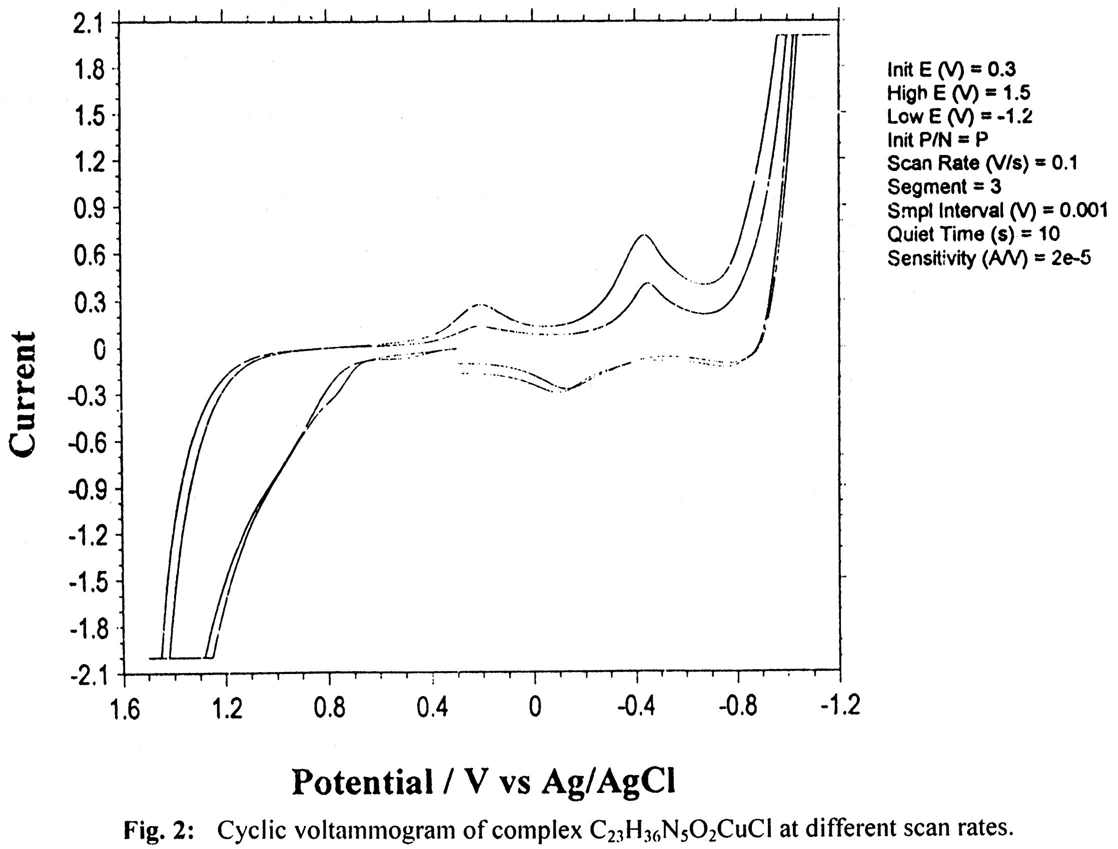

**Fig. 2:** Cyclic voltammogram of complex $C_{23}H_{36}N_5O_2CuCl$ at different scan rates.

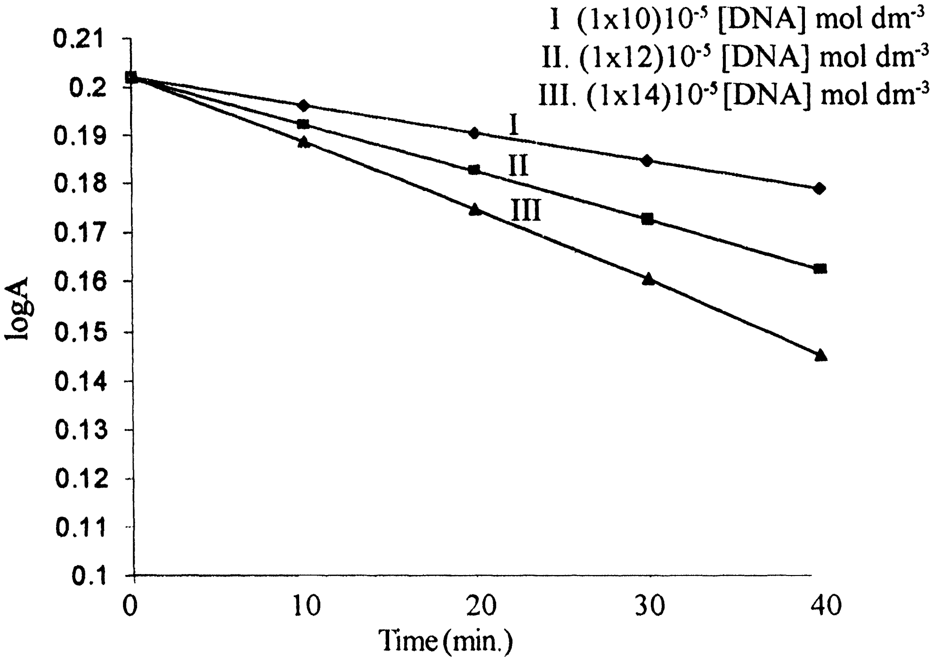

**Fig. 3:** Plot of log A versus time of the $C_{23}H_{36}N_5O_2CuCl$ at varying concentrations (10-18 × $10^{-5}$ mol $dm^{-3}$).

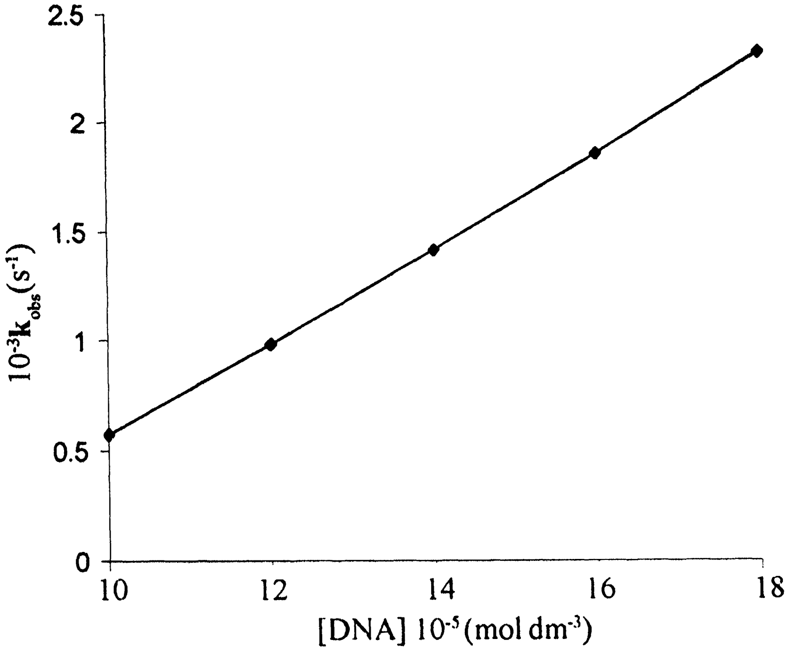

**Fig. 4:** Plot of $k_{obs}$ versus [DNA] of the $C_{23}H_{36}N_5O_2CuCl$ at varying concentrations (10-18 × $10^{-5}$ mol $dm^{-3}$).

### Kinetic Studies

The absorption spectra of calf thymus DNA, and $C_{25}H_{36}N_5O_2CuCl$ were recorded spectrophotometrically in MeOH/$H_2O$ (5:95) at $\lambda_{max}$ of calf thymus DNA at 260 nm and 30°C temperature. The absorption spectra of the complex $C_{25}H_{36}N_5O_2CuCl$ exhibit a soret band at 645 nm, attributed to the d–d transition. On interaction with calf thymus DNA, there is a red shift of 9 nm and large hypochromicity. Small changes in $\lambda_{max}$ and hypochromicities have been observed in some copper complexes upon interaction with calf thymus DNA[34,35]. Strong hypochromism and red shifts are usually attributed for intercalation /36/. The rate constant $k_{obs}s^{-1}$ values were calculated at varying concentration of calf thymus DNA by plotting logA *versus* time (Fig. 3) and the plot of the $k_{obs}$ versus [DNA] gave a straight line, suggesting a pseudo-first order dependence (Fig. 4). On the basis of the kinetic data, the following mechanism is proposed.

The observed rate law is

$$k_{obs} = k_1 k_2[DNA]/ (k_{-1}+ k_2) \quad (1)$$

### ACKNOWLEDGEMENT

We are grateful to the TWAS Italy for financial support through Ref. No. 98- 176/RG/CHE/AS and to the TIFR Mumbai for n.m.r. facilities .Thanks are due to the CDRI Lucknow for CHN data and IR analysis.

### REFERENCES

1. R. Tamilrason and D. R. McMillan, *Inorg. Chem.*, **29**, 2798 (1990).
2. A. Mazumdar and D. M. Perrin, *Chem. Rev.*, **93**, 2295 (1993).
3. K. A. Meadows, F. Liu, J. Sou, B. P. Hudson and D. R. Macmillan, *Inorg. Chem.*, **32**, 2919 (1993).
4. D. S. Sigman, *Acc. Chem. Res.*, **19**, 180 (1986).
5. D. S. Sigman, D. R. Graham, V. D'Aurora and A. M. Stern, *J. Biol. Chem.*, **254**, 12269 (1979).
6. D. S. Sigman and A. Spassky, *Biochemistry*, **24**, 8050 (1985).
7. W. K. Pogozelski and T. K. Tullius, *Chem. Rev.*, **98**, 1089 (1998).
8. C. J. Burrows and J. G. Miller, *Chem. Rev.*, **98**, 1109 (1998).
9. T. B. Thederahn, M. D. Kuwbara, T. A. Larsen and D.S. Sigman, *J. Am. Chem. Soc.*, **16**, 4941 (1989).
10. L. D. Williams, J. Thiverge and I. H. Goldberg, *Nucleic Acid Res.*, **16**, 11607 (1988).
11. J. M. Veal and R. L. Rill, *Biochemistry*, **30**, 1132 (1991).
12. R. Ren, P. Yang, W. Zheng and Z. Hua, *Inorg. Chem.*, **39**, 5454 (2000).
13. A. V. Terskikh, J. M. Leudoussal, R. Crameri, I. Fisch and J. P. Mach, *Proc. Natl. Acad. Sci, U.S.A.*, **94**, 1663 (1997).
14. D. A. Fancy, K. Melcher, S. A. Johnston and T. Khodadek, *Chem. Biol.*, **3**, 551 (1996).
15. L. Schmit, T. M. Bohanon, S. Denzinger, H. Ringsdorf and R. Tempw, Angew. *Chem. Int. Ed. Engl.*, **35**, 317 (1996).
16. W. Harada, T. Nojima, A. Shibayama, A. Ueda, H. Shindo and M. J. Chikira, *J. Inorg. Biochem.*, **64**, 273 (1996).
17. M. Chikira, M. Inoue, R. Nagane, W. Harada and H. Shindo, *J. Inorg. Biochem.*, **66**, 131 (1997).
18. R. Nagane, M. Chikira, M. Oumi, H. Shindo, and E. W. Antholine, *J. Inorg. Biochem.*, **78**, 243 (2000).

19. J. Murmur, *J. Mol. Biol.* **3**, 208 (1961).
20. P. Athappan and G. Rajgopal, *Transition Met. Chem.*, 22, 167 (1997).
21. J. N. Nwabueze, *Transition Met. Chem.* **22**, 123 (1997).
22. D. X. West, J. S. Ives, G. A. Bain, A. E. Liberta, J. V. Martinez, K. H. Ebert, S. H. Ortega, *Polyhedron*, **16**, 1895 (1997).
23. J. Kuncheria and K. K. Aravindakshan, *Synth. React. Inorg. Met. Org. Chem.*, **23**(9), 1469 (1993).
24. B. J. Hathaway, In: *Comprehensive Coordination Chemistry*; G. Wilkinson, R. D. Gillard, J. A. McCleverty (Eds.); Pergamon Press: Oxford, U. K. Vol. 5, p. 663-673, 1987.
25. A. B. P. Lever, *Inorganic Electronic Spectroscopy*, Elsevier, Amsterdam, p. 513-520, 1984.
26. M. D. Santana, G. Garcia, A. Refuete, G. Lopez, J. Casabo, A. Cabrero, E. Molins and C. Miravitlles, *Polyhedron*, 16, 3713 (1997).
27. M. D. Santana, A. Refuete, G. Garcia, G. Sanchez, G. Lopez, J. Casabo, E. Molins and C. Miravitlles, *Inorg. Chem. Acta*, **21**, 255 (1997).
28. K. Dey and K. K. Nandi, *Ind. J. Chem.*, **35A**, 766 (1996).
29. A. Bashal, M. Macpartlin, B. P. Murphy, H. R. Powell, and S. Walker. *J. Chem. Soc., Dalton Trans.*, 1383 (1994)
30. A. J. Bard and L. R. Faulkner, *Electrochemical Methods*, Wiley, New York, p. 219, 1980.
31. (a)M. Palaniandavar, T. Pandiyan, M. Laximinarayan and H. Monohar, *J. Chem. Soc., Dalton Trans.*, 455 (1995). (b). Rajesh and Mathur, *Polyhedron*, **17**, 2607 (1996).
32. S. Usha, and M. Palaniandavar, *J. Chem. Soc., Dalton Trans.*, 2277 (1994).
33. T. W. Weltch, and H. H. Thorp, *J. Phys. Chem.*, **100**, 13829 (1996).
34. R. F. Pasternack, E. J. Gibbs and J. J. Villfranca, *Biochemistry*, **22**, 2406 (1983).
35. S. Mahadevan and M. Palaniandavar, *Inorg. Chem.*, **37**, 693 (1990).
36. E . Long and J. K. Barton, *J. Acc. Chem. Res.*, **28**, 271 (1990).

